# Cost effectiveness and decision analysis for national airport screening options to reduce risk of COVID-19 introduction in Uganda, 2020

**DOI:** 10.1186/s12962-024-00548-x

**Published:** 2024-05-12

**Authors:** Geofrey Amanya, Michael L. Washington, Daniel Kadobera, Migisha Richard, Alex Ndyabakiira, Julie Harris

**Affiliations:** 1 Uganda Public Health Fellowship Program, Ministry of Health, Kampala, Uganda; 2 Division of Preparedness and Emerging Infectious Diseases, US Centers for Disease Control and Prevention, Atlanta, USA; 3 Division of Global Health Protection, US Centers for Disease Control and Prevention, Kampala, Uganda; 4 Infectious Diseases Institute, College of Health Sciences, Makerere University, Kampala, Uganda

**Keywords:** Screening, COVID-19, Pandemic, Cases averted, Cost-effectiveness, Policy

## Abstract

**Introduction:**

Early during the COVID-19 outbreak, various approaches were utilized to prevent COVID-19 introductions from incoming airport travellers. However, the costs and effectiveness of airport-specific interventions have not been evaluated.

**Methods:**

We evaluated policy options for COVID-19-specific interventions at Entebbe International Airport for costs and impact on COVID-19 case counts, we took the government payer perspective. Policy options included; (1)no screening, testing, or mandatory quarantine for any incoming traveller; (2)mandatory symptom screening for all incoming travellers with RT-PCR testing only for the symptomatic and isolation of positives; and (3)mandatory 14-day quarantine and one-time testing for all, with 10-day isolation of persons testing positive. We calculated incremental cost-effectiveness ratios (ICERs) in US$ per additional case averted.

**Results:**

Expected costs per incoming traveller were $0 (Option 1), $19 (Option 2), and $766 (Option 3). ICERs per case averted were $257 for Option 2 (which averted 4,948 cases), and $10,139 for Option 3 (which averted 5,097 cases) compared with Option I. Two-week costs were $0 for Option 1, $1,271,431 Option 2, and $51,684,999 Option 3. The per-case ICER decreased with increase in prevalence. The cost-effectiveness of our interventions was modestly sensitive to the prevalence of COVID-19, diagnostic test sensitivity, and testing costs.

**Conclusion:**

Screening all incoming travellers, testing symptomatic persons, and isolating positives (Option 2) was the most cost-effective option. A higher COVID-19 prevalence among incoming travellers increased cost-effectiveness of airport-specific interventions. This model could be used to evaluate prevention options at the airport for COVID-19 and other infectious diseases with similar requirements for control.

**Supplementary Information:**

The online version contains supplementary material available at 10.1186/s12962-024-00548-x.

## Introduction

The increase over the past few decades in global air travel provides countless opportunities for infections to spread, both to passengers on the plane and the community after arrival [1]. Since the advent of the SARS-CoV-2 pandemic, multiple interventions have been employed around the world to limit or slow the introduction of SARS-CoV-2 from air travellers. These have included physical distancing, hand sanitizing, and masking at airports and throughout travel, pre-and-post-travel testing, temperature and symptom screening on entry, symptom monitoring among incoming travellers, quarantine policies for travellers, and border closures [2]. These strategies have varied across nations, with varying levels of sustainability, consideration of the resources of health care systems, and acceptability to the community [3]. While no country has succeeded in maintaining an entirely COVID-19-free state, interventions aimed at travellers have almost certainly delayed or reduced the impact of the epidemic in multiple countries, including in Uganda [4- 6].

Uganda has one major international airport (Entebbe International Airport (EBB)) through which most international air travel starts or ends in the country. Although it has three smaller airports that receive short-range flights from neighbouring countries, 98% of incoming international air travellers enter Uganda through EBB [7]. Since the first case of COVID-19 in Uganda was reported on March 21, 2020, a number of control measures were implemented at the airport to reduce transmission risk, including installation of hand sanitizer stations, queue separators to keep people from crowding while they waited in lines, and a 14-day mandatory institutional quarantine for all travellers from high-risk countries [8]. Countries were initially categorised by the Ugandan government into high and low risk based on the active and cumulative number of COVID-19 cases reported by the country [9].

Due to vaccinations, increased global immunity, and other interventions, COVID-19 might cease to be a significant travel-associated threat over time. It is also possible that new variants will perpetuate the travel-associated threats of COVID-19. Regardless, future epidemics during which airport-specific interventions will again become relevant are all but certain: screening for Ebola Virus Disease was still in place at EBB when COVID-19 screening began in February 2020 [10]. Despite this, only one such study involving South Africa have examined the cost-effectiveness of preventive interventions at airports for COVID-19 [11]. We compared the costs and cost-effectiveness of different hypothetical policy options for COVID-19-specific interventions at EBB to guide decision-making by national stakeholders during this and future epidemics.

## Methods

### Study design

We used a decision analysis approach. We performed a literature review using PubMed (Medline) and other sources, including the World Health Organization (WHO) website, country reports, and Uganda-specific governmental reports to obtain data to inform our assumptions; where no documented data were available, we obtained expert recommendations. A decision tree was developed to compare different options for screening and testing at the airport. To estimate the number of secondary cases for each policy option, we multiplied the respective reproductive number (R) by the number of cases that reach the community. We calculated incremental cost effectiveness ratios (ICERs) expressed as US$ per additional COVID-19 case averted for two strategies when compared to the base case strategy. Expected costs and cases were calculated for each strategy. We conducted a sensitivity analysis for the most uncertain variables.

### Perspective and cost data

The Uganda Ministry of Health (MOH) was the payer for the airport-related interventions in Uganda early during the epidemic, including supporting the testing and mandatory quarantine for Ugandan travellers as well as isolation of infected persons [12]. As a result, we took the government payer perspective. Cost data were obtained from the operations and finance departments of the Ministry of Finance [13] (Table 1). All costs were calculated for 14 days of operation, considering all persons entering the country via EBB over a two-week period (estimated based on traveller volume before the pandemic). We did not include infrastructure or utility costs such as buildings, electricity, and water bills.

### Policy options

#### Policy option 1: no intervention

Under this policy, there was no screening at the airport and no required testing, quarantine, or isolation for incoming airport travellers. Travellers could enter quarantine optionally or seek testing, but not through a MOH/airport-affiliated program.

#### Policy option 2: mandatory symptom screening for all incoming travellers, testing only the symptomatic

Under this policy, all 67,500 incoming travellers would be screened at the airport for symptoms consistent with COVID-19 (with the prevalence of symptomatic persons dependent on the array of symptoms chosen for screening). Any incoming travellers identified as symptomatic would undergo required testing. Persons testing positive would enter mandatory government-sponsored isolation. All isolated persons would be released after 10 days of isolation without a second test.

#### Policy option 3: mandatory quarantine, symptom monitoring and testing for all incoming travellers

Under this policy, all 67,500 incoming travellers would be tested, and if they test negative, they would undergo 14 days of mandatory institutional quarantine (using hotels, schools, or other institutional settings able to accommodate large numbers of people); persons testing positive would be isolated 10 days.

### Assumptions

We made multiple assumptions based on the literature where data were available and obtained expert opinions where data were unavailable (Table 1). We did not include the cost of treatment for illness in travellers, because it has not been a government expense during the pandemic in Uganda.

**Table 1:** Assumptions used to evaluate policy options in decision tree of national airport screening options for COVID-19, 2021

Epidemiologic parameters	Value	Sensitivity analysis (Min, Max)	References/ Assumptions
Number of reported travellers coming through EBB airport in two weeks during pre-pandemic period*	67,500	---	[7]
Prevalence of SARS-CoV-2 infection among travellers	0.05	(0.01, 0.10)	assumption
Proportion of infected travellers with COVID-19-like symptoms at / shortly after arrival	0.63	---	[29]
Proportion of travellers with symptoms detected by the screeners through temperature and symptom checks	0.40	(0.05, 0.75)	assumption
Proportion of infected travellers with COVID-19-like symptoms at / shortly after arrival who seek a test	0.60	---	assumption
Proportion of uninfected travellers symptomatic with COVID-19-like symptoms at / shortly after arrival^†^	0.022	---	[5, 30]
Probability that uninfected (true-negative) persons who enter quarantine are infected by others in quarantine who have undetected (false-negative) infections (Option 3)	0.005	---	assumption
Probability that symptomatic infected persons detected on entry remain infectious at 10 days	0.02	---	[14]
Probability that asymptomatic infected persons detected on entry remain infectious at 10 days	0.01	---	[31]
Probability that uninfected (false-positive) person in isolation becomes infected from others (true-positives) (Options 2 & 3)^¥^	0.50	---	GA, unpublished data
Proportion of uninfected travellers with COVID-19-like symptoms who later seek a test ^**¶**^	0.60	---	assumption
Reproductive number (R) for infected, symptomatic persons	2.60	---	[32]
Reproductive number (R) for infected, asymptomatic persons	1.40	---	Uganda MoH, unpublished data
Probability that travellers with COVID-19-like symptoms on arrival are sent for testing (Option 2)	0.92	---	assumption
Probability that uninfected travellers will be symptomatic with COVID-19-like symptoms and warrant a COVID-19 test	0.01	---	assumption
Sensitivity of test (RT-PCR)	0.92	(0.50, 1.00)	[33]
Specificity of test (RT-PCR)	0.98	---	[34]
**Cost parameters**	Cost ($USD)		[13]
Disinfection supplies	$3	---	
Staff (health worker) per person for two weeks (*n* = 8)	$400	---	
Gowns per person per day	$10	---	
Face masks per person per day	$3	---	
Gloves per person per day	$23	---	
Gumboots per person (one-time cost)	$6	---	
PCR test per person	$65	($10, $100)	
Printing costs per person	$3	---	
Handwashing (alcohol-based hand sanitiser) per person per day	$3	---	
Handheld infrared thermometer	$50	---	
Airport transfer to and from quarantine/isolation per person	$10	---	
Room and board per person per day	$50	---	

*Values stated as a whole number, remaining assumptions expressed as proportions/probabilities

^†^Value applies to incoming travellers for Policy Options 2&3

^**¶**^This value applies to incoming travellers for Policy Options 1&2

^**¥**^This value applies to unpublished COVID-19 outbreak data in home-based care setting in southwestern Uganda

For Policy Option 3, we assumed that all persons sent to quarantine adhered to quarantine for the appropriate time for the full quarantine period, and that all persons testing positive from Options 2 and 3 were isolated appropriately for the full isolation period. As we did not specify a second test before release from quarantine or isolation, we assumed that 1% of asymptomatic persons and 2% of symptomatic persons were still infectious after being released from isolation (Options 2 and 3) or quarantine (Option 3) [14]. We also assumed a small risk (0.5%) of persons without infection becoming infected in quarantine from other persons placed in quarantine (Option 3). In addition, we included a small risk of infection (0.5%) to others from persons that had tested false negative and were placed in quarantine (Option 2), and a large risk (50%) of infection of persons who had tested false positive and were placed in isolation with other persons with infection (Options 2 and 3). All of those infected in isolation were assumed to be infectious on release. For those who remained infectious at release, we assume their R would correspond to the symptomatic status upon arriving to the airport.

### Outcomes

The primary outcome measure was the ICER per COVID-19 case averted for each option considering one generation of spread, compared to Option 1. Secondary outcomes included the expected cost of each option per incoming traveller at EBB, cases identified through the intervention, cases that end up in the community due to failure to be identified in the intervention activities, case counts after a single generation of cases from infected travellers entering the community, and the expected estimated costs per policy option.

## Results

At a prevalence of 5% for SARS-CoV-2 infection among the incoming travellers, 3,375 infected persons would enter the airport in a two-week period for each option. The expected cost per traveller was more than 40 times higher for Option 3 vs. Option 2 ($766 vs. $19) (Table 2). Compared to Option 1, Option 2 averted 4,948 COVID-19 cases, and Option 3 adverted 5,097 cases. This resulted in an ICER of $257 per case averted for Option 2 and $10,139 for Option 3 (Table 2).

**Table 2:** Cost-effectiveness analysis for the three policy options over a two-week timeframe, Uganda 2020

Outcome	Option 1: No symptom screening or testing	Option 2: Mandatory symptom screening for all, testing only the symptomatic	Option 3: Mandatory quarantine and testing for all
Infected travellers entering at airport	3,375	3,375	3,375
Identified through intervention (all persons who test true positive in isolation)	0 ^†^	1,965	3,105
Infected travellers who enter community	3,375	1,472	1,010
First-generation cases resulting from Infected travellers who enter community	7,277	2,329	2,179
Expected cost per traveller	$0	$19	$766
Total costs for intervention (US$)	$0	$1,271,431	$51,684,999
Cases averted compared with Option 1	0	4,948	5,097
ICER cost/case averted*	-	$257	$10,139

*ICER: Incremental cost-effectiveness ratio, ICER = (Costs2-Costs1)/(Effectiveness2-Effectiveness1)

^†^ Cases would be identified for Option 1, but not under government perspective

### Cost drivers

The primary cost drivers for Option 3, compared with Option 2, involved the costs of quarantine, which comprised 87% of the total costs for Option 3 (Supplementary Table 1).

## Sensitivity analysis

### Variation in ICER by infection prevalence

Infection prevalence among incoming travellers had a major impact on the ICER. However, the cost-effectiveness of Option 3 was much more dependent than Option 2 on prevalence, especially at varying SARS-CoV-2 prevalence among travellers (Supplementary Table 2).

Supplementary Table 2 highlights the Impact of infection prevalence on expected costs for mandatory symptom screening for all, testing only the symptomatic and mandatory quarantine, symptom monitoring and testing for cost-effectiveness of national airport COVID-19 screening, 2021. Value of base model in bold.

**Fig. 1:**
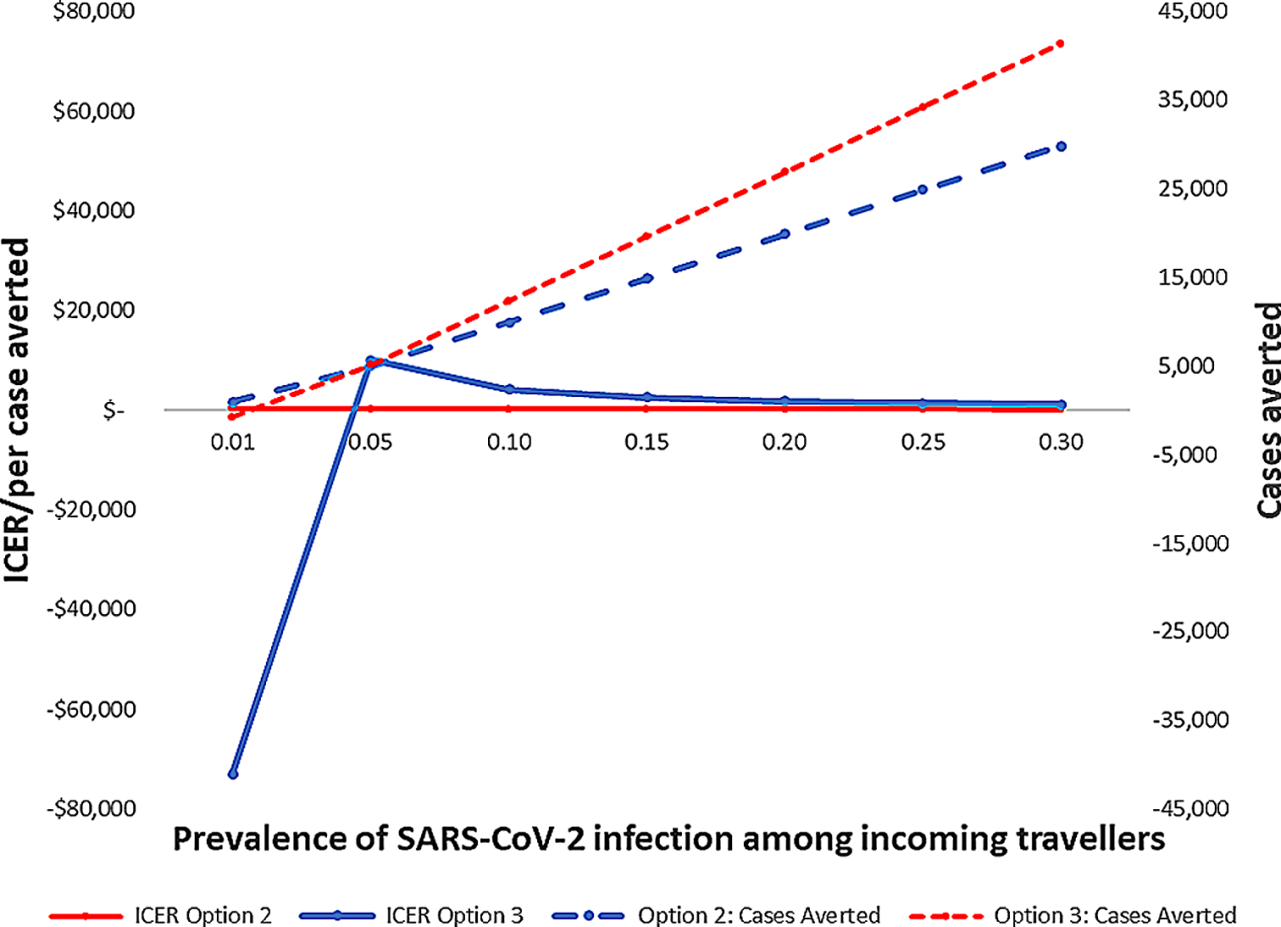
Sensitivity analysis for Option 2&3 compared to option 1 for national Airport programme, COVID-19, 2021

For Option 1, all 675 infected individuals entered the community. For Option 3, those 675 individuals entered either quarantine or isolation. Although most of the 675 individuals left uninfected, they infected enough people such that 1,007 individuals are infected by the time they leave quarantine or isolation. Option 3 put more people in the community to infect others than Option 1. This made Option 3 strongly dominant (e.g., less effective and more costly), and thus generated a negative ICER when Option 3 was compared to Option 1. For the other prevalences, the opposite was true (more cases enter the community in Option 1 than Option 3), creating a positive ICER. While Option 2 was always more cost-effective in terms of ICER per case averted, the ICER of the two intervention options became more similar at high prevalence (Fig. 1).

Figure 1, highlights Sensitivity analysis for Option 2 (mandatory symptom screening for all, testing only the symptomatic) and Option 3 (mandatory quarantine, symptom monitoring, and testing for all), compared to option 1 in decision tree of national airport screening options for COVID-19, 2021. ICER: incremental cost-effectiveness ratio. At 0.01 prevalence, 675 people arrive to the airport infected.

### Variation in ICER by diagnostic test sensitivity

The ICERs for Options 2 and 3 decreased with increasing sensitivity of the diagnostic test. However, the cost-effectiveness of Option 3 was always weakly dominated by Option 2, even at very high sensitivity of the diagnostic test (Supplementary Table 3). That is, Option 3 was always the most effective, but also the costliest by far. Test cost was not a major driver of cost-effectiveness within the range of costs considered (Supplementary Table 4).

Supplementary Table 3 highlights the Impact of diagnostic test sensitivity on ICER (Incremental Cost-Effectiveness Ratio) for Option 2 (mandatory symptom screening for all, testing only the symptomatic incoming travellers at the airport point of entry) and Option 3 (mandatory quarantine, symptom monitoring, and testing for all incoming travellers at the airport point of entry), evaluation of cost-effectiveness of national airport screening options for COVID-19, 2021.

This table highlights the Impact of diagnostic test cost on Incremental Cost-Effectiveness Ratio (ICER) for mandatory symptom screening for all, testing only the symptomatic and mandatory quarantine, symptom monitoring and testing for all, evaluation of cost-effectiveness of national airport screening options for COVID-19, 2021.

### Variation in ICER by symptom ‘detectability’

Because Option 2 relies so heavily on the detection of symptoms, we varied the probability that symptoms would be detected or admitted to by the passenger, from 5 to 75%. At higher symptom detection rates, the ICER for Option 2 decreased almost by 50%. Although 29,342 infected travellers were detected at 75% and 1,956 at 5% (data not shown), the increased cost of isolation of all the symptomatic passengers kept the ICER relatively stable (Supplementary Table 5).

This table highlights the Impact of variations in symptom detection among symptomatic persons for mandatory symptom screening for all, testing only the symptomatic and mandatory quarantine, symptom monitoring and testing for all on cost per case averted, evaluation of cost-effectiveness of national airport screening options for COVID-19, 2021. Value of base model in bold.

## Discussion

We evaluated the cost-effectiveness of multiple airport screening policy options for COVID-19 using a decision tree. The three policy options considered in this study are consistent with modest (Option 2) and intensive (Option 3) practices used during similar public health emergencies in the past [15]^,^ [16]. Among the policy options considered, mandatory symptom screening for all incoming airport travellers and testing only the symptomatic (Option 2) was more cost-effective than mandatory quarantine and testing for all (Option 3). This was primarily due to the high total costs of mandatory quarantine for all travellers in Option 3. Overall, the results from this evaluation suggest that the modest Option 2 could reduce secondary spread of SARS-CoV-2 infections from incoming airport travellers in the most cost-effective manner.

Over the time period considered, the cost-effectiveness of our interventions was modestly sensitive to the prevalence of COVID-19 in travellers, diagnostic test sensitivity, symptom detection, and testing costs. The intensive Option 3 became more cost-effective at a higher prevalence of infection among incoming travellers. At a very high prevalence among incoming travellers, the cost-effectiveness of Option 3 became very close to Option 2. The high costs of Option 3 – nearly $52 M USD per two weeks in Uganda - might all but rule it out in some settings, such as in Uganda, where relatively limited resources combined with porous borders with five neighbouring countries make it nearly impossible to implement a true ‘zero-COVID-19’ approach. However, for some countries this might be the preferred route: at different points in time, Australia required mandatory hotel quarantine for incoming travellers, New-Zealand imposed similar measures for mandatory quarantine for 20 days, and Hong Kong banned passengers from more than 150 nations from transiting through its airport [17, 18]. Countries with fewer borders and the ability to implement stricter border control might select an option like Option 3.

The sensitivity of symptom screening varies, depending on multiple factors, and any approach that depends on subjectively-measured symptoms is likely to be less sensitive than one that uses objectively-measured symptoms. Travellers may be reluctant to admit to symptoms, or even take steps to avoid symptom detection such as using cough suppressants or antipyretic medications. As a result, most airports will be limited for practical reasons to persons willing to self-report their symptoms or mass screening technologies, such as thermal scanners. Indeed, at the Entebbe airport, thermal scanners have been used intermittently since January 2019 for all incoming travellers to screen for Ebola virus disease, and consistently since March 2020 to screen for COVID-19 [19]^,^ [20]. The first case identified in Uganda was identified by temperature screening [5]. However, such approaches may be rendered ineffectual if travellers do not want to admit to symptoms, or use antipyretics to mask fevers [21]. In addition, many persons do not have a fever or other symptoms when infected with SARS-CoV-2, making infections difficult to identify and reducing the effectiveness of temperature or symptom screening [22]. Surprisingly, we found that varying the rates of symptom detection (from 5 to 75%) had minimal impact on the cost-effectiveness of Option 2 compared with Option 1. This is not because of a minor change in infection detection, but rather due to the increased costs associated with isolating more cases, as well as the lower assumed prevalence of infection in incoming travellers (5%) [23]. At a higher prevalence of infection, or if the costs of isolation were borne by the travellers instead of the government, the cost-effectiveness of Option 2 might increase as symptom detection increased.

Under the conditions considered, testing represented a surprisingly small proportion of overall costs for Options 2 and 3. This was reflected in the relative insensitivity of changes in test costs on the cost-effectiveness of either option. As a country considers similar airport-specific interventions for COVID-19 or for other diseases in the future, it may be worth considering modifications to the quarantine and isolation components of such programs to increase their cost effectiveness, rather than focusing on new testing modalities. While lower costs of diagnostics are critical to identify individual cases, they may not have much impact on screening programs that involve government-payer isolation and quarantine. Of course, should the cost of any of these interventions be placed with the travellers, all of the options described would be considerably more cost-effective from a government payer perspective.

The expected costs per incoming traveller were only $19 for Option 2, more than 40-fold lower than the expected costs for Option 3. The expected costs for Option 2 were highly sensitive to changes in prevalence, while the expected costs for Option 3 were not. This is largely due to the fact that all incoming persons are quarantined regardless of infection prevalence, and this component comprises the bulk of Option 3 costs. The expected cost for Option 2 is quite low, especially at a lower prevalence of infection. Compared to the cost of airline tickets, this cost is negligible and could potentially be placed on the traveller. The International Health Regulations suggest that health measures that benefit a traveller may incur a charge, not to exceed the cost of the service [24]. A cost may be bound on the passenger via the airline ticket with a “health screening charge” [25]. This airline ticket fee could be similar to the passenger facilitation or security charge used elsewhere [26].

This model can appropriately represent situations in which the primary risk from a disease is coming from incoming airport travellers. That is, while it is likely useful early in a COVID-19 or COVID-19-like epidemic, when the main risk comes from incoming international travellers, it may not be as relevant if there are widespread community infections, at which point it cannot effectively prevent internal spread. The primary purpose of airport interventions should be to delay or reduce the introduction of community infection and gain time to implement other public health measures, such as improving the preparedness of healthcare systems and public health prevention measures [27]. However, airport interventions are not likely to have a major impact during Phase 4 (sustained community level transmission) of the epidemic [28].

Our evaluation had some limitations. First, there are few consistent data available on many of our key assumptions, such as proportion of persons who are symptomatic (for which real-world estimates depend on the completeness of testing in an exposed population), true sensitivity or specificity of different symptom screening approaches or sensitivity and specificity of diagnostic tests (due to challenges with a ‘gold standard’ to help confirm who was genuinely infected), and the SARS-CoV-2 infection reproductive values for symptomatic and asymptomatic persons (for which real-world estimates depend on the completeness of testing of contacts). Second, we assumed complete compliance with quarantine and no bypassing of airport screening interventions, which may not be realistic. Third, we assumed that issues that are common to many low-resource settings, such as stockouts of supplies or power failures, would not affect our outcomes, which may similarly not represent reality in our setting. Fourth, we assumed a constant rate of infection during isolation and quarantine no matter the infection prevalence. Fifth, the model itself considers the detection of cases among incoming travellers and the single-generation impact of minimizing transmission from these travellers as the primary goal; none of the options considered are appropriate for a ‘zero-COVID-19’ approach as some countries have taken. Finally, we did not include the infrastructure and utility costs. These costs would have increased the cost and cost-effectiveness ratio, but our final recommendations would likely be the same.

This model simulates realistic, real-world airport-specific interventions that have been used in the past, including in Uganda [10]. It supports discussions about the benefits and costs of airport-specific screening activities. These are often adopted in a reactive manner rather than with a cost-effectiveness mindset. Findings from this study provide valuable evidence that can be used by decision-makers to help assess the costs and benefits of alternative airport screening strategies for COVID-19 or similar infectious diseases.

## Conclusion

Mandatory symptom screening for all travellers and testing only the symptomatic is more cost-effective than the other options considered for preventing the introduction of COVID-19 into the general population. Early in the emergence of a highly pathogenic COVID-19-like pandemic, screening all incoming travellers, testing those symptomatic, and isolating the persons testing positive should be considered.

### Funding and disclaimer

This project was supported by the President’s Emergency Plan for AIDS Relief (PEPFAR) through the US Centers for Disease Control and Prevention Cooperative Agreement number GH001353–01 through Makerere University School of Public Health to the Uganda Public Health Fellowship Program, MoH. Its contents are solely the responsibility of the authors and do not necessarily represent the official views of the US Centers for Disease Control and Prevention, the Department of Health and Human Services, Makerere University School of Public Health, or the Uganda MoH. The staff of the funding body provided technical guidance in the design of the study, ethical clearance and collection, analysis, and interpretation of data and in writing the manuscript.

### Electronic Supplementary Material

Below is the link to the electronic supplementary material.

Supplementary Material 1

## Data Availability

The datasets used and/or analysed in the during this study are available from the corresponding author upon request.

## Declarations

### Data sharing

This modelling study involved the use of published or publicly available data. The data used and the sources are described in the manuscript and appendix. No primary data were collected for this study. Model flowcharts are in the appendix.

## Abbreviations

ICER: Incremental Cost-Effectiveness Ratios
CI: Confidence interval
COVID-19: Coronavirus disease 2019
SARS-CoV-2: Severe Acure respiratory Syndrome
MOH: Uganda Ministry of Health
EBB: Entebbe International Airport
RT-PCR: Reverse transcriptase polymerase chain reaction
PEPFAR: President’s Emergency Plan for AIDS Relief
R: Reproductive number

## References

[1] Mangili A, Tines, Vindenes, Gendreau M. Infectious risks of Air Travel. Microbiol Spectr. Sep. 2015;3(5). 10.1128/MICROBIOLSPEC.IOL5-0009-2015. 10.1128/microbiolspec.IOL5-0009-2015 26542037

[2] Chinazzi M et al. Apr., The effect of travel restrictions on the spread of the 2019 novel coronavirus (COVID-19) outbreak, *Science (80-.)*, vol. 368, no. 6489, pp. 395–400, 2020, 10.1126/SCIENCE.ABA9757. 10.1126/science.aba9757 PMC7164386 32144116

[3] Kringos D et al. Managing COVID-19 within and across health systems: why we need performance intelligence to coordinate a global response, *Heal. Res. Policy Syst* 2020 181, vol. 18, no. 1, pp. 1–8, Jul. 2020, 10.1186/S12961-020-00593-X. 10.1186/s12961-020-00593-x PMC7358993 32664985

[4] Yaya S, Otu A, Labonté R. Globalisation in the time of COVID-19: repositioning Africa to meet the immediate and remote challenges, *Glob. Heal* 2020 161, vol. 16, no. 1, pp. 1–7, Jun. 2020, 10.1186/S12992-020-00581-4. 10.1186/s12992-020-00581-4 PMC7312111 32580728

[5] Migisha R, et al. Early cases of SARS-CoV-2 infection in Uganda: epidemiology and lessons learned from risk-based testing approaches – March-April 2020. Global Health. Dec. 2020;16(1):114. 10.1186/s12992-020-00643-7. 10.1186/s12992-020-00643-7 PMC7686950 33239041

[6] Anderson RM, Heesterbeek H, Klinkenberg D, Hollingsworth TD. How will country-based mitigation measures influence the course of the COVID-19 epidemic? *Lancet*, vol. 395, no. 10228, pp. 931–934, Mar. 2020, 10.1016/S0140-6736(20)30567-5. 10.1016/S0140-6736(20)30567-5 PMC7158572 32164834

[7] Uganda Bureal of Statistics_ (Statistical_Abstract). Statistical abstract 2020. Accessed: Jun. 17, 2021. [Online]. Available: https://www.ubos.org/wp-content/uploads/publications/11_2020STATISTICAL__ABSTRACT_2020.pdf.

[8] Uganda US-E. COVID-19 information | U.S. Embassy in Uganda. https://ug.usembassy.gov/covid-19-information-page/ (Accessed Jul 29, 2021).

[9] Amatya B, Khan F. Rehabilitation Response in Pandemics, *Am. J. Phys. Med. Rehabil*, vol. 99, no. 8, pp. 663–668, Aug. 2020, 10.1097/PHM.0000000000001477. 10.1097/PHM.0000000000001477 PMC7268860 32452879

[10] Aceng JR et al. Uganda’s experience in Ebola virus disease outbreak preparedness, 2018–2019, *Glob. Heal* 2020 161, vol. 16, no. 1, pp. 1–12, Mar. 2020, 10.1186/S12992-020-00548-5. 10.1186/s12992-020-00548-5 PMC7081536 32192540

[11] Reddy KP (2020). Cost-effectiveness of public health strategies for COVID-19 epidemic control in South Africa: a microsimulation modelling study. medRxiv.

[12] Press Releases | African Development Bank -. Building today, a better Africa tomorrow. https://www.afdb.org/en/news-and-events/press-releases (Accessed Jul 09, 2021).

[13] Uganda MF. Ministry of Finance, Planning and Economic Development | #DoingMore. https://www.finance.go.ug/ (Accessed Jul 09, 2021).

[14] Walsh KA (2020). The duration of infectiousness of individuals infected with SARS-CoV-2. J Infect.

[15] Martin G, Boland M. Planning and preparing for public health threats at airports, *Glob. Heal* 2018 141, vol. 14, no. 1, pp. 1–5, Mar. 2018, 10.1186/S12992-018-0323-3. 10.1186/s12992-018-0323-3 PMC5842601 29514664

[16] Alonso TD. An airport operations proposal for a pandemic-free air travel. J Air Transp Manag. Jan. 2021;90:101943. 10.1016/J.JAIRTRAMAN.2020.101943. 10.1016/j.jairtraman.2020.101943 PMC7544451 33052179

[17] James Griffiths and Amy Woodyatt. Hong Kong announces new border closures, as China’s coronavirus death toll overtakes SARS., Hong Kong, Jan. 2020. Accessed: Feb. 09, 2022. [Online]. Available: https://www.cnn.com/2020/02/03/asia/wuhan-coronavirus-update-intl-hnk/index.html.

[18] Radio New Zealand., Prime-minister-jacinda-ardern-reveals-move-to-level-1-from-midnight, New Zealand, Jun. 2020. Accessed: Feb. 09, 2022. [Online]. Available: https://web.archive.org/web/20200608035311/https://www.rnz.co.nz/news/political/418524/prime-minister-jacinda-ardern-reveals-move-to-level-1-from-midnight.

[19] Observer. No one can escape coronavirus screening at Entebbe - Aviation. Accessed: Jul. 19, 2021. [Online]. Available: https://observer.ug/news/headlines/63900-no-one-can-escape-coronavirus-screening-at-entebbe-aviation.

[20] Uganda_CAA. Uganda intensifies Ebola screening at international airport – Civil Aviation Authority. https://caa.go.ug/uganda-intensifies-ebola-screening-at-international-airport/ (Accessed Jul 19, 2021).

[21] Bwire GM, Paulo LS. Coronavirus disease-2019: is fever an adequate screening for the returning travelers? *Trop. Med. Heal* 2020 481, vol. 48, no. 1, pp. 1–3, Mar. 2020, 10.1186/S41182-020-00201-2. 10.1186/s41182-020-00201-2 PMC7061485 32165854

[22] Bielecki M, et al. Air travel and COVID-19 prevention in the pandemic and peri-pandemic period: a narrative review. Travel Med Infect Dis. Jan. 2021;39:101915. 10.1016/J.TMAID.2020.101915. 10.1016/j.tmaid.2020.101915 PMC7655026 33186687

[23] Philip Dollard M. MPH, Francisco Alvarado-Ramy, Risk Assessment and Management of COVID-19 Among Travelers Arriving at Designated U.S. Airports, January 17–September 13, 2020,Morbidity and Mortality Weekly Report (MMWR), Nov. 2020. [Online]. Available: https://www.cdc.gov/mmwr/volumes/69/wr/mm6945a4.htm?s_cid=mm6945a4_w. 10.15585/mmwr.mm6945a4 PMC7660668 33180758

[24] ., Wilson Kumanan, Halabi S, The International Health Regulations. and Gostin Lawrence O., (2005), the threat of populism and the COVID-19 pandemic, *Glob. Heal. 2020 161*, vol. 16, no. 1, pp. 1–4, Jul. 2020, 10.1186/S12992-020-00600-4. 10.1186/s12992-020-00600-4 PMC7386836 32723370

[25] Tabares DA. An airport operations proposal for a pandemic-free air travel. J Air Transp Manag. Jan. 2021;90:101943. 10.1016/J.JAIRTRAMAN.2020.101943. 10.1016/j.jairtraman.2020.101943 PMC7544451 33052179

[26] Gillen D, Morrison WG. Aviation security: Costing, pricing, finance and performance, *J. Air Transp. Manag*, vol. 48, pp. 1–12, Sep. 2015, 10.1016/J.JAIRTRAMAN.2014.12.005.

[27] Kadowa I. Using evidence and analysis for an adaptive health system response to COVID-19 in Uganda in 2020 in association with training and Research Support Centre in the Regional Network for Equity in Health in East and Southern Africa (EQUINET) EQUINET Case study paper with support from the. Open Society Policy Centre; 2020.

[28] World Health Organization, Pandemic influenza preparedness and response: a WHO guidance document. World Health Organization,. Geneva. 2009. Accessed: Jan. 12, 2022. [Online]. Available: https://apps.who.int/iris/bitstream/handle/10665/44123/9789241547680_eng.pdf. 23741778

[29] Li F, et al. Household transmission of SARS-CoV-2 and risk factors for susceptibility and infectivity in Wuhan: a retrospective observational study. Lancet Infect Dis. May 2021;21(5):617–28. 10.1016/S1473-3099(20)30981-6. 10.1016/S1473-3099(20)30981-6 PMC7833912 33476567

[30] N. OT et al., SARS-CoV-2 Infection among Travelers Returning from Wuhan, China, *N. Engl. J. Med*, vol. 382, no. 15, pp. 1476–1478, Apr. 2020, 10.1056/NEJMC2003100. 10.1056/NEJMc2003100 PMC7121487 32163698

[31] Oran DP, Topol EJ. Prevalence of asymptomatic SARS-CoV-2 infection. Ann Intern Med. Feb. 2021;174(2):286–7. 10.7326/L20-1285. 10.7326/L20-1285 33587872

[32] Rahman B, Aziz IA, Khdhr FW, Mahmood DF. Preliminary estimation of the Basic Reproduction Number of SARS-CoV-2 in the Middle East, 10.2471/BLT.20.251561.

[33] Stites EC, Wilen CB. The Interpretation of SARS-CoV-2 Diagnostic Tests, *Med (New York, N.y.)*, vol. 1, no. 1, p. 78, Dec. 2020, 10.1016/J.MEDJ.2020.08.001. 10.1016/j.medj.2020.08.001 PMC7441939 32864639

[34] Padhye NS. Reconstructed diagnostic sensitivity and specificity of the RT-PCR test for COVID-19, *medRxiv*, p. 2020.04.24.20078949, Feb. 2021, 10.1101/2020.04.24.20078949.

