# Supplementary material for: Cost effectiveness and decision analysis for national airport screening options to reduce risk of COVID-19 introduction in Uganda, 2020
Source: Cost Eff Resour Alloc. 2024 May 12;22:40. doi: 10.1186/s12962-024-00548-x (PMC11089758; doi:10.1186/s12962-024-00548-x)
Supplement: Supplementary file 1 — Supplementary Material 1 [file 12962_2024_548_MOESM1_ESM.docx]

**Supplementary Table 1:** Cost drivers in decision tree of national airport screening options for COVID-19, 2021

| Item | Option 2:  Mandatory symptom screening for all, testing only the symptomatic | | | Option 3:  Mandatory quarantine  and testing for all | | |
| --- | --- | --- | --- | --- | --- | --- |
|  | Cost $USD |  | (%) | Cost $USD |  | (%) |
| **Total Cost** | **1,271,432** |  |  | **51,684,999** |  |  |
| Isolation | 1,034,609 |  | (81) | 2,281,837 |  | (4) |
| Quarantine | 0 |  | (0) | 45,003,629 |  | (87) |
| Screening | 6,096 |  | (1) | 6,096 |  | (0) |
| Testing | 230,726 |  | (18) | 4,393,438 |  | (9) |

**Supplementary Table 2:** Impact of infection prevalence on expected costs for Option 2&3, for national Airport program, 2021.

| Prevalence | Expected value for Option 2:  Mandatory symptom screening for all, testing only the symptomatic | Expected value for Option 3:  Mandatory quarantine and testing for all |
| --- | --- | --- |
| 0.01 | $ 5 | $ 773 |
| **0.05** | **$ 19** | **$ 766** |
| 0.10 | $ 36 | $ 757 |
| 0.15 | $ 53 | $ 748 |
| 0.20 | $ 70 | $ 740 |
| 0.25 | $ 87 | $ 731 |
| 0.30 | $ 104 | $ 722 |

**Supplementary Table 3:** Impact of diagnostic test sensitivity on ICER for Option 2&3, for national Airport program, 2021.

| Test Sensitivity | ICER for Option 2:  Mandatory symptom screening for all, testing only the symptomatic | ICER for Option 3:  Mandatory quarantine and testing for all |
| --- | --- | --- |
| 0.50 | $ 302 | $ 10,198 |
| 0.60 | $ 286 | $ 10,184 |
| 0.70 | $ 274 | $ 10,170 |
| 0.80 | $ 265 | $ 10,156 |
| 0.90 | $ 258 | $ 10,142 |
| 1.00 | $ 253 | $ 10,128 |

**Supplementary Table 4.** Impact of diagnostic test cost on ICER for Option 2&3 for national Airport program, 2021.

| Diagnostic test cost (in USD) | ICER for Option 2:  Mandatory symptom screening for all, testing only the symptomatic | ICER for Option 3:  Mandatory quarantine and testing for all at arrival |
| --- | --- | --- |
| 10 | $ 218 | $ 9,411 |
| 20 | $ 225 | $ 9,543 |
| 30 | $ 232 | $ 9,676 |
| 40 | $ 239 | $ 9,808 |
| 50 | $ 246 | $ 9,941 |
| 60 | $ 253 | $ 10,073 |
| 70 | $ 261 | $ 10,206 |
| 80 | $ 268 | $ 10,338 |
| 90 | $ 275 | $ 10,470 |
| 100 | $ 282 | $ 10,603 |

**Supplementary Table 5.** Impact of variations in symptom detection on ICER for Option 2&3, national Airport program, 2021.

| Symptom detection (among symptomatic) | ICER for Option 2:  Mandatory symptom screening for all, testing only the symptomatic | ICER for Option 3:  Mandatory quarantine and testing for all |
| --- | --- | --- |
| 5% | $ 583 | $ 14,861 |
| **40%** | **$ 272** | **$ 11,592** |
| 75% | $ 253 | $ 9,520 |
